# Formulation, characterization and cellular toxicity assessment of a novel bee-venom microsphere in prostate cancer treatment

**DOI:** 10.1038/s41598-022-17391-w

**Published:** 2022-08-02

**Authors:** Samia E. El-Didamony, Reham I. Amer, Ghada H. El-Osaily

**Affiliations:** 1 Zoology and Entomology Department, Faculty of Science, Al-Azhar University, Youssef Abbas St. off Mostafa Elnhhas, 6th District, Nasr City, Cairo 11751 Egypt; 2 Department of Pharmaceutics and Pharmaceutical Technology, Faculty of Pharmacy, Al-Azhar University, Youssef Abbas St. off Mostafa Elnhhas, 6th District, Nasr City, Cairo 11751 Egypt; 3 Department of Pharmaceutics, Faculty of Pharmacy, October University of Modern Sciences and Arts (MSA), 26 July Mehwar Road intersection with Wahat Road, 6th of October, Giza, 12611 Egypt

**Keywords:** Cancer, Medical research

## Abstract

Bee venom (B.V.) is a toxin produced naturally by honey bees with several toxic and therapeutic efficacies. It is used in the treatment of different cancer kinds like renal, hepatic, and prostate cancer. Due to its protein nature, it is degraded in the upper gastrointestinal tract. Colon-targeted drug delivery systems represent a useful tool to protect B.V. from degradation and can be administered orally instead of I.V. infusion and traditional bee stinging. In the present study, B.V. loaded enteric-coated cross-linked microspheres were prepared by emulsion cross-linking method. Percentage yield, entrapment efficiency %, swelling degree, and in-vitro release are evaluated for prepared microspheres. Free B.V., optimized microspheres formula (F3), and doxorubicin cytotoxic effects were tested by MTT assay. Results concluded that free B.V. was more effective against the growth of human prostate adenocarcinoma (PC3) cells followed by optimized microspheres than doxorubicin. But both free B.V. and doxorubicin have a cytotoxic effect on normal oral epithelial cells (OEC). According to flow cytometric analysis, the optimized microsphere formula induced apoptosis and reduced necrosis percent at IC_50_ concentration. Furthermore, microspheres did not affect the viability of OEC. These results revealed that microspheres have a degree of specificity for malignant cells. Therefore, it seems that this targeted formulation could be a good candidate for future clinical trials for cancer therapy.

## Introduction

Prostate cancer (PC) is considered the most common male cancer in developed countries. This neoplasm is diagnosed in a large number of middle-aged males^1^. It is usually asymptomatic in its early stages and not revealed until reached its advanced stages. After diagnosis, the rate of survival is low, about 32%. There are different treatment options for prostate cancer such as surgery, radiation therapy, hormone therapy, and chemotherapy^2^. A small number of chemotherapeutic drugs are used in PC treatment. Resistance to chemotherapy is a major problem in the treatment of cancer^3^. The main impedance of these chemotherapeutic drug delivery is their aqueous poor solubility and toxicity because of lacking target specificity^4^ and many of them cause nonspecific cell damage^5^.

However, an ongoing endeavor to improve therapy and reduce the death rate by different research groups brings new hopes. It was later deduced that most new chemotherapeutic drugs are derived from natural sources^6^. Bee venom (B.V.) is a normal biologically active protein complex consisting of melittin, phospholipase A2, apamin, and hyaluronidase with great therapeutic efficacy. Many effects of B.V. have been reported recently, like necrosis, cytotoxicity, effects on proliferation, apoptosis induction, and inhibition of growth of several cancer cell types^7^. Even though B.V. proteins can treat a lot of cancer cells like prostate, lung, renal, hepatic, mammary cells, and leukemia cells^8–13^ but, till now it has still exhibited many drawbacks, especially, when administered by intravenous infusion. It shows a short plasma half-life in addition to the inability to determine the exact dose after stinging by bees. On the other hand, it is degraded upon oral administration due to the presence of pepsin and trypsin, with subsequent very low oral bioavailability (only 5.22%). However, the use of B.V. has shown adverse effects on normal cells as reported by^14–16^. Thus, there is still a need for more studies that can eliminate or even decrease the cytotoxic effect of B.V. and enhance its therapeutic effect in the target organ. Targeted drug delivery (TDD) has been developed as a powerful strategy for the treatment of cancer because of the higher delivery of medications, to the tumor site with maximum protection from the extracellular environment^17^. Colon drug delivery is considered one of a useful tool to deliver proteins and peptide drugs that are degraded by digestive enzymes of the stomach and small intestine orally. That allows high protein concentrations to reach the colon. In addition to that, there is a longer retention time for the colonic contents (up to 5 days), and drug absorption is facilitated by the colonic mucosa. All of that makes the colon a perfect site for drug delivery^18^. Microspheres are types of microparticles in the form of free-flowing powder with less than 200 µm in particle size. They are composed of biodegradable polymers like chitosan. They are used mainly as controlled release drug carriers. There are different methods such as spray congealing, polymerization, phase separation, and double emulsion have been used mainly in preparing them^19^.

In an attempt to improve cancer therapeutic protocols, this study was undertaken to develop an optimized formulation to increase the related drug anticancer properties as well as reduce their systemic side-effects and evaluate its antitumor effect against PC3. Additionally; the development of cross-linked chitosan enteric-coated microspheres was evaluated as a controlled drug carrier system for effective delivery of oral B.V. over an extended period of time with a remarkable degree of specificity for malignant cells.

## Materials

Chitosan (medium molecular weight), Glutaraldehyde, Potassium phosphates and dimethyl sulfoxide (DMSO) were purchased from Sigma Aldrich (St Louis, MO, USA). RPMI-1640, penicillin–streptomycin, trypsin–EDTA, fetal bovine serum (FBS) and glutamine were obtained from Gibco BRL (Grand Island, NY, USA). Coomassie Brilliant Blue G-250, 3-(4,5-dimethylthiazol-2-yl)-2,5-diphenyltetrazolium bromide (MTT), and Phosphoric acid (85%) were obtained from Merck Co. (Darmstadt, Germany). Acetone, glacial acetic acid, liquid paraffin, n-hexane and ethanol were purchased from ADWIC, (Egypt). Eudragit S 100 (ES 100) was obtained from Evonik (Darmstadt, Germany). Span^®^ 80 and bovine serum albumin (BSA) were kindly supplied by Fluka (Egypt).

## Methods

### Collection of B.V.

B.V. was collected from healthy workers of the honey bee, Apis mellifera (L.) according to Ref.^20^ using the electro-stimulation method. Briefly, the electric shock device (VC-6F model from Apitronic Services, 9611, No. 4 Road, Richmond, B.C., Canada) comprises a frame with wire electrodes installed in parallel to each other. The frames were mounted on the top or under the hive and then connected to an electro-stimulator. The electrical impulses stimulated the bee workers to sting through latex, which was placed on a glass plate. We transferred the glass plate carefully to the laboratory, in which the venom was dried at an ambient temperature. Then, we used a sharp scraper to scrape off the dry venom. After that, fresh bee venom was stored in dark glass tubes at a temperature of – 4 °C until needed. 1 mg of B.V. was diluted in 1 mL of distilled water to prepare a stock solution of the venom. Centrifugation (15,000*g*, 5 min.) was conducted at 25 °C after vertex. The supernatant was filtered using a 0.2 membrane filter and kept at − 40 °C in the dark.

### Preparation of B.V. loaded cross-linked chitosan microspheres

Three formulations of B.V. loaded cross-linked chitosan microspheres with different B.V.: polymer ratios were prepared (Table 1). Firstly, chitosan was added to 1% aqueous glacial acetic acid with continuous stirring overnight by a magnetic stirrer. Then B. V. was added to the prepared solution while mixing. The formed mixture was injected after that into liquid paraffin containing span 80 using a syringe with mechanical stirring for 30 min. to form w/o emulsion. Glutaraldehyde (5%) was then added dropwise and the mixture was left for 7 h to allow cross-linking^21^. The formed microspheres were collected by centrifugation, washed with acetone, and finally dried at 50 °C in a hot air oven.

**Table 1:** Composition of different B.V. loaded cross-linked chitosan microspheres.

Formulations	B.V.:polymer
F_1_	1:1
F_2_	1:2
F_3_	1:4

### Coating of B.V. loaded cross-linked chitosan microspheres

The coating process was performed using solvent evaporation method with ES 100. The prepared microspheres were initially dispersed in ES 100 solution containing ethanol and acetone. Then it was poured in a mixture of span 80 and liquid paraffin with subsequent agitation for 3 h at room temperature. The mixture was then filtered, washed with n-hexane, and finally overnight freeze-dried^19^.

### Characterization of B.V. loaded cross-linked chitosan-coated microsphere

#### Percentage yield (%)

B.V. loaded cross-linked chitosan-coated microspheres are weighed and the % yield is calculated using the following equation^19^:

$$\% {\text{Yield}} = Actual\;weight\;of\;the\;product \times 100/{\text{Total}}\;{\text{weight}}\;{\text{of}}\;{\text{excipient}}\;{\text{and}}\;{\text{drug}}$$

The test was done in triplicate and the results are represented as percentage yield mean ± SD (n = 3).

### Entrapment efficiency (% EE)

To calculate the amount of B.V. entrapped inside the prepared coated microspheres. Phosphate buffer saline (PBS) pH 7.4 was added to a known amount of B.V. loaded cross-linked chitosan-coated microspheres. The formed mixture was vigorously stirred with a mechanical stirrer for 24 h. Centrifugation was then applied and the supernatant was collected to determine B.V. content. Finally, the amount of B.V. was successfully measured spectrophotometrically at ʎ_max_595 using Bradford protein assay method^22^. Bovine Serum Albumin (BSA) was used as a protein concentration standard. The entrapment efficiency is calculated using the following equation^19^:

$$\% {\text{EE}} = {\text{Practical}}\;{\text{drug}}\;{\text{content}} \times 100/{\text{Theoretical}}\;{\text{drug}}\;{\text{content}}.$$

EE% was carried out in triplicate, data were represented as mean ± SD (n = 3).

### Degree of swelling

Place weighed amount of different B.V. loaded cross-linked chitosan coated microspheres in enzyme-free simulated intestinal fluid pH 7.4, leave it till swelling in the dissolution apparatus at 37 °C ± 0.5 °C. Then the treated microspheres were dried between filter paper and then weighted. Changing in weight is still measured until equilibrium is reached. The following equation is used to calculate the swelling ratio^19^:

$$\mathbf{S}\mathbf{R}=\frac{\mathrm{Wg }-\mathrm{ Wo}}{\mathrm{ Wo}},$$

where SR is the swelling ratio, Wo is the Initial weight, Wg is the Final weight.

The test was done in triplicate and the results are represented as mean ± SD (n = 3).

### Scanning electron microscopy (SEM)

SEM (JSM 5300, JOEL, Japan) was used to detect the morphological structure of the prepared cross-linked chitosan-coated microspheres. Firstly, the microspheres were coated using a sputter coater with gold and then dried using an ion beam-based system with a single vacuum. For imaging by SEM, computer i-scan 2000 software was used^23^.

### In vitro drug release study

An accurately weighed amount of B.V. loaded cross-linked chitosan-coated microspheres from each formulation were placed in tea bags and immersed in a pH progressive media of 37 °C ± 0.5 °C, 100 rpm. The study was done using dissolution test apparatus paddle type. The tea bag tying has been assisted by the stringed paddle. Gastrointestinal transit conditions can be simulated by changing the pH of the dissolution medium at different time intervals. The pH of the dissolution medium was maintained at 1.2 with 0.1 N HCl for 2 h. By adjusting the pH to 7.4, the release study was observed and continued for another 3 h. After that, the pH was adjusted to pH 6.8 and continued for 24 h^19^. Finally, the samples were taken from the dissolution medium at different time intervals and the drug release rate was effectively measured spectrophotometrically at ʎmax595 using Bradford protein assay method. Each formula was estimated in triplicate and the results are represented as mean ± SD (n = 3).

### Kinetic study

The in vitro release data was fitted to first-order, zero-order kinetics and Higuchi equations and also to general exponential function: M_t_/M_∞_ = kt^n^, where M_t_/M_∞_ represents solute release regarding to conditions of equilibrium; the exponent of diffusion (n) is the characteristics of the release mechanism and k is used for drug and polymer properties^23^**.**

### In-vitro cytotoxic effect of free B.V., B.V. loaded cross-linked chitosan- coated microspheres and doxorubicin

#### Cell culture

Human Prostate adenocarcinoma (PC3) has been used as a cancer cell line while oral epithelial cells (OEC) were used as a normal cell line during this investigation. Both cells were obtained from the American Type Culture Collection (Manassas, VA) and grown in RPMI-1640 medium supplemented with 10% fetal bovine serum (FBS), 2 mM l-glutamine, 1 mM sodium pyruvate, and penicillin/streptomycin (100 U/mL). Cell lines were maintained at 37 °C and 5% CO_2_.

### MTT assay

Cell viability was assessed using the MTT reduction test to determine the effects of free B.V. and B.V. loaded cross-linked chitosan- coated microspheres as well as doxorubicin as positive control on PC3 and OEC cells. In brief, cells (1 × 105 cells/mL) were seeded in 96 well micro-titer plates (Nunc-Denmark) at a concentration of 1 × 105 cells/mL (100 µL/well) and incubated until a complete monolayer sheet developed. After the monolayer sheet of cells was formed, the growth media was decanted and the cells were treated with (1.93, 3.87, 7.75, 15.5, 31, and 62 µg/mL) of both B.V. & doxorubicin and 100 mg/mL of B.V. loaded cross-linked chitosan coated microspheres in the volume of 100 μL⁄well. The control was added to saline of equal volume. Plates were incubated at 37 °C and 5% CO_2_ atmospheric conditions for 24 h. After, that the media were removed, plates were washed with phosphate-buffered saline (PBS), and the cells were incubated with 50 µL/well of (3-(4,5-Dimethylthiazol-2-yl)-2,5-ditetrazolium bromide (MTT) solution for 4 h, then DMSO solution was added as 0.05 mL/well. Finally, the absorbance of each well was measured at 570 nm wavelength using an ELIZA reader.

The viability percent was calculated as follows:

$$\mathrm{Viability \; \%}=\frac{\mathrm{Mean \; OD\; Treated }}{\mathrm{ Mean \; OD \; Control}}\times 100,$$

where, OD is optical density.

The IC_50_ is the concentration of tested material required to inhibit 50% of cell growth, and the value was calculated by an online tool^24^**.**

### Morphological analysis

Cancer PC3 cells were seeded in 12-well plates containing RPMI-1640 supplemented with 10% fetal calf serum (FCS) at a density of 5 × 105 cells/well and incubated for 24 h. Then the media were removed and the cells were treated with (1.93, 3.87, 7.75, 15.5, 31, and 62 µg/mL) of both B.V. & doxorubicin and 100 mg/mL of B.V. loaded cross-linked chitosan coated microspheres and incubated for 24 h. After that, the cells were fixed with 4% paraformaldehyde and stained with 0.1% crystal violet at room temperature, decolorized with 33% acetic acid. Morphological changes in treated cells were observed and compared to untreated cells using an inverted phase-contrast microscope (Helmut Hund GmbH, Wetzlar, Germany).

### Detection of apoptosis by flow cytometric assay

In order to examine the type of cell death induced by tested formula (B.V. loaded cross-linked chitosan coated microspheres) in PC3 cells, flow cytometric analysis was performed using the Annexin V-FITC Apoptosis Detection Kit I (BD Biosciences) according to the manufacturer’s protocol. PC3 cells were treated with IC50 concentration of B.V. loaded cross-linked chitosan coated microspheres and incubated for 24 h. The treated and untreated cells as control were trypsinized and pelleted down, centrifuged (1000*g*, 5 min, 24 °C), washed with cold PBS, and centrifuged (1000*g*, 5 min, 24 °C). Then, 5 µL of Annexin-V-FITC and 5 µL of propidium iodide were added and maintained in the dark for15 minutes. Finally, the samples were analyzed using a flow cytometer (CyFlow SL, Partec-Germany) at 488 nm to quantify the proportion of live, dead, apoptotic and necrotic cells. The Navios software (Beckman Coulter) was used to analyse flow cytometry data. Experiments were performed independently in triplicate.

### Statistical analysis

The examinations were performed in three replicates and the data were represented as Mean ± standard deviation using Sigma plot 12.5 and Microsoft office 365. Student’s t-test was used to analyze the difference between the experiment group and the control group in the flow cytometry assay. P < 0.05 was considered to indicate a statistically significant difference.

## Results and discussion

### Percentage yield (%)

**Figure 1:**
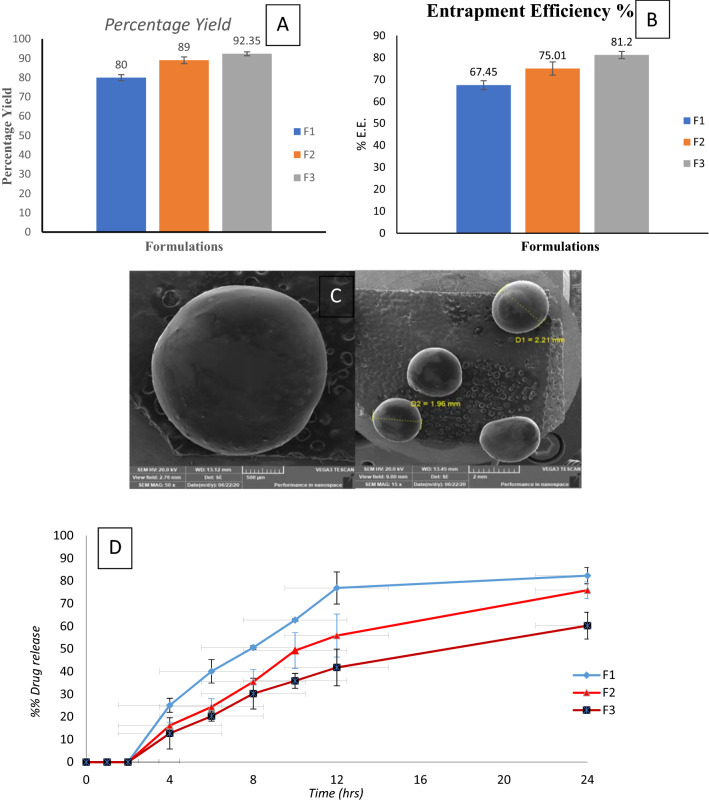
Characterization of B.V. loaded cross-linked chitosan-coated microspheres: (**A**) Percentage yield, (**B**) Entrapment Efficiency %, (**C**) SEM image of surface morphology (**D**) In vitro release profile in different pH media.

The percentage yield of B.V. loaded cross-linked chitosan-coated microspheres formulations is represented in Fig. 1A. The percentage yield of F1, F2, and F3 were 80, 89, and 92.35% respectively. We can easily detect that by increasing B.V.: chitosan ratio, the percentage yield (%) of the prepared B.V microspheres was markedly increased.

### Entrapment efficiency (% EE)

Entrapment efficiency is supposed to be an important factor as insufficient entrapment causes an initial burst drug release and that prevents the sustained release feature of the microspheres. Also, an insufficient entrapment affects the required therapeutic dose, which should be available to attain the intended therapeutic effect. Entrapment efficiency of BV loaded cross-linked chitosan-coated microspheres was determined in Fig. 1B^25^. The results showed that the entrapment efficiency increased with an increase in drug: polymer ratio. This observation might be due to the increase in the aqueous phase viscosity as a result of B.V: polymer ratio increase), which has led to stabilizing the formed microspheres and also hinder drug flow throughout the hardening phase^26^**.**

### Degree of swelling

The physiological swelling capacity of the prepared B.V microspheres in the medium was determined and the results are given in Table 2. No obvious swelling was observed with B.V. loaded cross-linked chitosan-coated microspheres. That is, confirming the better resistance of ES100-coated microspheres in the upper GIT to prevent inflation and subsequent release at non-target locations^19^.

**Table 2:** Degree of swelling of different B.V-cross-linked chitosan coated microspheres.

B.V. coated microspheres formulation	Degree of swelling ± SD
F1	0.09 ± 0.031
F2	0.05 ± 0.015
F3	0.1 ± 0.061

### Scanning electron microscopy (SEM)

Microsphere morphology examination was basic to diagnose microsphere’s structure and realize its behavior. The morphological images of the prepared B.V-cross-linked chitosan-coated microspheres are presented in Fig. 1C, where, the microspheres seemed spherical with smooth morphology, and no aggregated microspheres were observed. The stabilized spherical shapes were detected to confirm the malleability of the formed microspheres, as a result of the cross-linking with glutaraldehyde. This cross-linking plays a significant role in microspheres surface morphology^27^.

### In vitro release study

The in-vitro release profile of B.V. from its different cross-linked chitosan-coated microspheres formulations was studied in different pH media for consecutive 24 h. Assuredly, as shown in Fig. 1D, B.V. has not been released from its cross-linked chitosan-coated microspheres in pH 1.2 for 2 h. Whereas B.V. has been started to release upon placed in pH 7.4 and the release continued at a higher rate upon placing in pH 6.8. As ES100 coat is ionized and its integrity was affected. Eudragit has carboxyl groups which are ionized in neutral and alkaline mediums. This ionization is disturbing ES100 structure resulting in the release of B.V.^25^. As shown, the cumulative percentage of the B.V release from its different formulations after 24 h. was in the range of 60.26–82.35% which revealed a slow rate. This slow-release rate is due to chemical cross-linking between chitosan and glutaraldehyde. Inter- and intramolecular cross-linking reactions occur through covalent bond formation^28^. F3 showed the slowest release pattern among different B.V microspheres formulations. It is valuable to say that the drug release (%) was obviously decreased with the increase in the chitosan quantity in the prepared microspheres, this observation might be a result of higher polymer matrix density in F3, that increasing the length of the diffusional pathway and lowering the release of drug from its matrix. In addition, lower concentrations of polymer form smaller particles which provide a larger surface area exposed to the dissolution medium^19^.

### Kinetics study

Results displayed in Table 3 show the in vitro drug release kinetic data and Korsmeyer–Peppas equation data. The values of the release exponents were 0.8740 to 1.1721. Based on these data, both F1 and F3 formulations exhibited Non-fikian diffusion, while the F2 formulation showed Super case II transport. Similarly, F1 and F3 formulations showed high (r) values for Higushi diffusion plots indicating the drug release followed Higushi diffusion release kinetics^29^. While F2 showed high (r) values for that First-order plots indicating the drug release followed first-order kinetics.

**Table 3:** Kinetic data of different B.V. loaded cross-linked chitosan coated microspheres.

Formula	Zero order		First order		Higushi diffusion		Korsmeyer–Peppas			Possible kinetics order and mechanism of the drug releaser
	r	k	r	k	r	K	r	K	n	
F_1_	0.8561	0.0440	0.9136	0.0012	0.9145	2.5748	0.9970	0.0006	0.9900	Diffusion, Non-fikian diffusion
F_2_	0.9438	0.0485	0.9873	0.0011	0.9764	2.7464	0.9968	0.0001	1.1721	First order kinetics, Super case II transport
F_3_	0.9630	0.0378	0.9893	0.0007	0.9898	2.1289	0.9749	0.0006	0.8740	Diffusion, Non-fikian diffusion

The chosen orders are in under line.

From all previous results, F3 was chosen as the optimized formula to evaluate the B. V. cytotoxic effect. It had a higher percentage yield, higher EE%, slowest in vitro release pattern, and showed diffusion release kinetics.

### In-vitro cytotoxic effect using MTT assay

**Figure 2:**
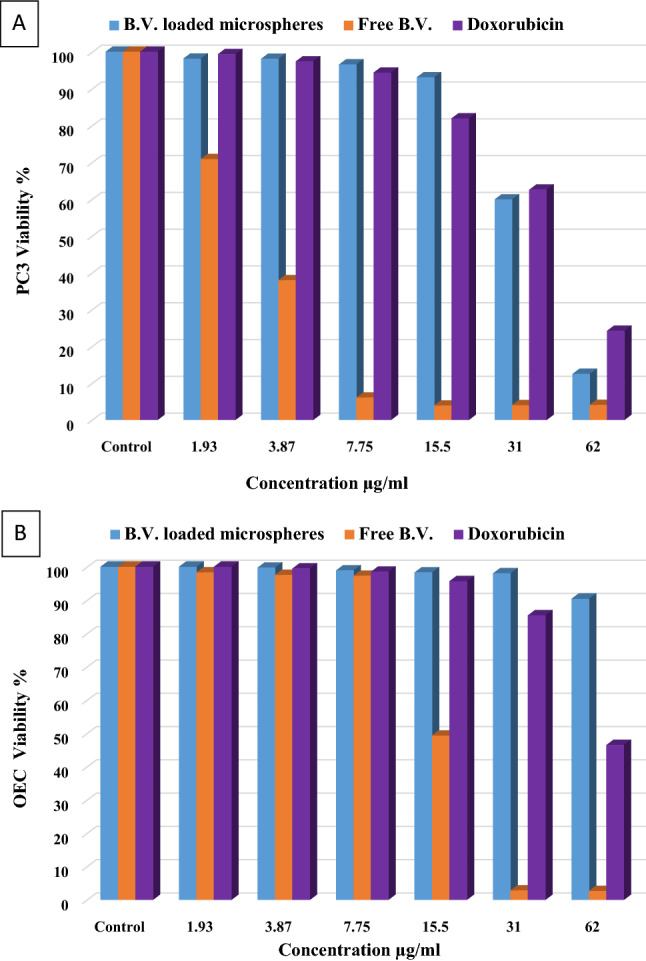
Effect of free B.V., B.V. loaded cross-linked chitosan coated microspheres and doxorubicin on (**A**) PC3 cancer cells (**B**) OEC normal cells.

To evaluate the cytotoxic effect of free B.V., B.V. loaded cross-linked chitosan-coated microspheres, and doxorubicin as positive control on cell growth of PC3 and OEC, cell viability was tested by MTT assay. Figure 2 shows that free B.V., B.V. loaded cross-linked chitosan-coated microspheres and doxorubicin inhibited cell proliferation (decreased the number of viable cells) of PC3 cells compared to the control cells in a concentration-dependent way. Twenty-four hrs. treatment of free B.V., B.V. loaded cross-linked chitosan-coated microspheres and doxorubicin inhibited PC3 cell growth with IC_50_ values of 3.87 ± 0.61, 34.57 ± 0.67, and 40.82 ± 1.005 μg/mL, respectively. Although doxorubicin is considered one of the anti-cancer drugs currently used to treat different types of cancer, it has the least effect on the cancer cells in this study, and it also has a toxic effect on the normal OEC viability cells with IC50 value of 49.33 ± 0.88 μg/mL. Also, free B.V. decreased the percentage of viable normal OEC cells in a concentration-dependent (Fig. 2B) with IC_50_ value of 15.54 ± 0.48 µg/mL. Nevertheless, treatment with microspheres has no toxic effect on the normal OEC viability cells. These results revealed that microspheres have a degree of specificity for malignant cells^30^. Data presented in Table 4 demonstrated the comparison between the 50% inhibitory concentrations of treatment with free B.V., B.V. loaded cross-linked chitosan-coated microspheres, and doxorubicin on the different used cell lines (PC3 and OEC).

**Table 4:** IC_50_ value of tested materials (Free B.V., B.V. loaded cross-linked chitosan coated microspheres and doxorubicin) on PC3 and OEC.

Tested materials	Normal cells (OEC)	Cancer cells (PC3)
	Mean ± SD	
Free B.V	15.54 ± 0.48	3.87 ± 0.61
B.V. loaded cross-linked chitosan coated microspheres	–	34.57 ± 0.67
Doxorubicin	49.33 ± 0.88	40.82 ± 1.005

### Morphological analysis

**Figure 3:**
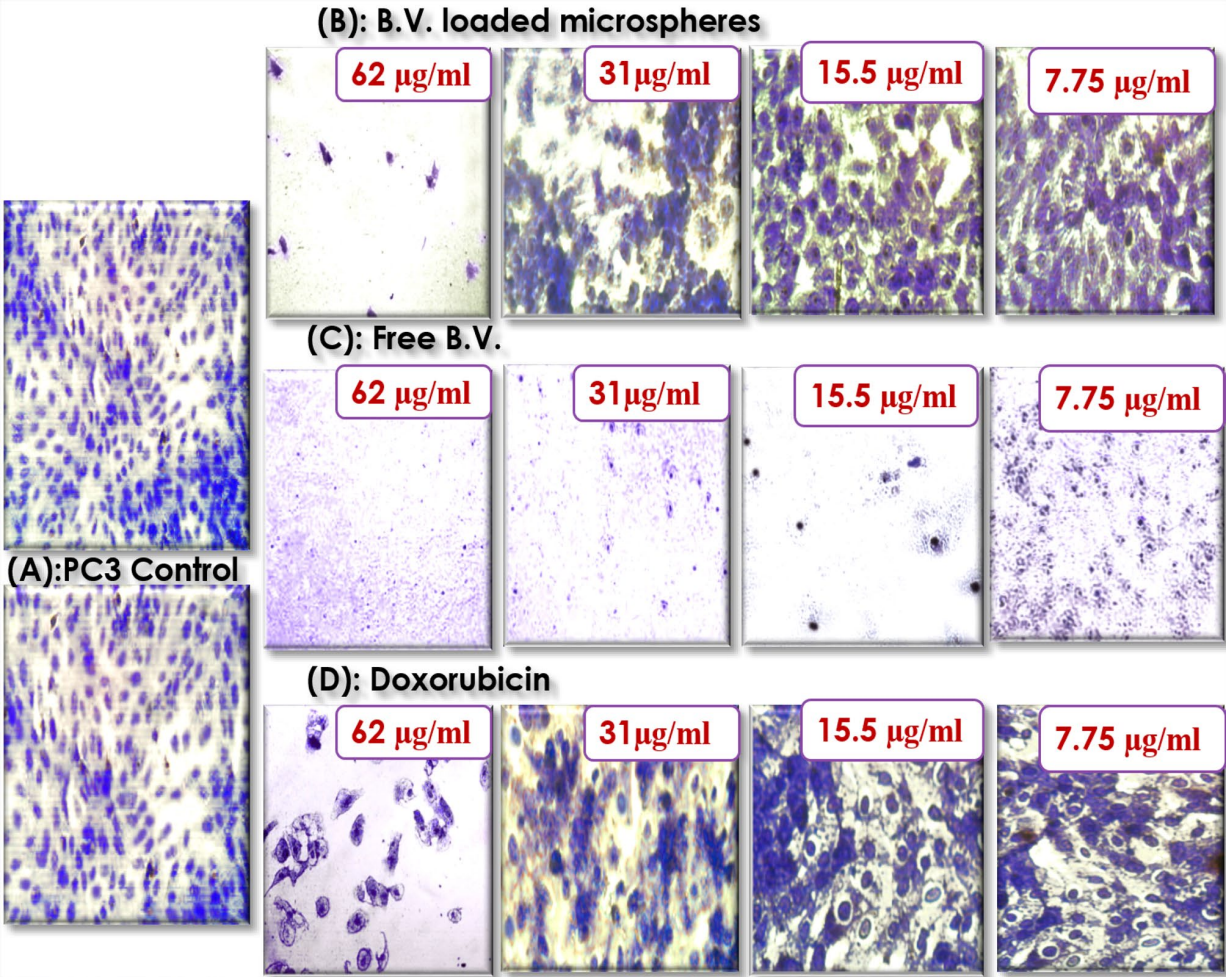
Microscopic images of PC3 cells with crystal violet staining before (**A**) PC3 control and after treatment with different concentrations of (**B**) B.V. loaded cross-linked chitosan coated microspheres (**C**) Free B.V. (**D**) Doxorubicin.

Crystal violet staining showed that incubation of prostate cancer cells with free B.V. and 31 µg/mL of B.V. loaded cross-linked chitosan coated microspheres and doxorubicin for 24 h reduced the number of viable cells compared to the control cells in a concentration-dependent. The results indicated typical effect of MTT assay where free B.V. was the most effective against PC3 cells followed by B.V. loaded cross-linked chitosan coated microspheres then doxorubicin. As shown in Fig. 3 the major morphological changes included cell shrinkage (Fig. 3C) and disorder in the cell structure under exposure to free B.V. in case of B.V. loaded cross-linked chitosan-coated microspheres and doxorubicin morphological changes were the formation of apoptotic bodies, cytoplasm condensation and cells lost their shape and became swollen (Fig. 3B,D) as compared to control cells (Fig. 3A).

The capability of venom-loaded nanoparticles in preventing the growth of cancer cells was also reported as B.V. loaded chitosan nanoparticles improved the effect of bee venom against HePG2 and PC3^31^. The IC_50_ values of free B.V. treatment were 20 and 49.4 g/mL for HePG2 and PC3, respectively. These values were decreased to reach 16.5 and 36.08 µg/mL in the case of B.V. loaded chitosan nanoparticles of HePG2 and PC3, respectively and it was concluded that the combination of B. V. with chitosan nanoparticles enhanced the inhibitory effect on colon cancer cells more than treatment with bee venom alone^10^. Also, It was reported that the cytotoxicity of a protein derived from the venom of Indian cobra *Naja kaouthia* when loaded on gold nanoparticles (GNPs) was more evident than native protein on leukemic cells (U937 and K562)^32^.

### Detection of apoptosis using flow cytometry

**Figure 4:**
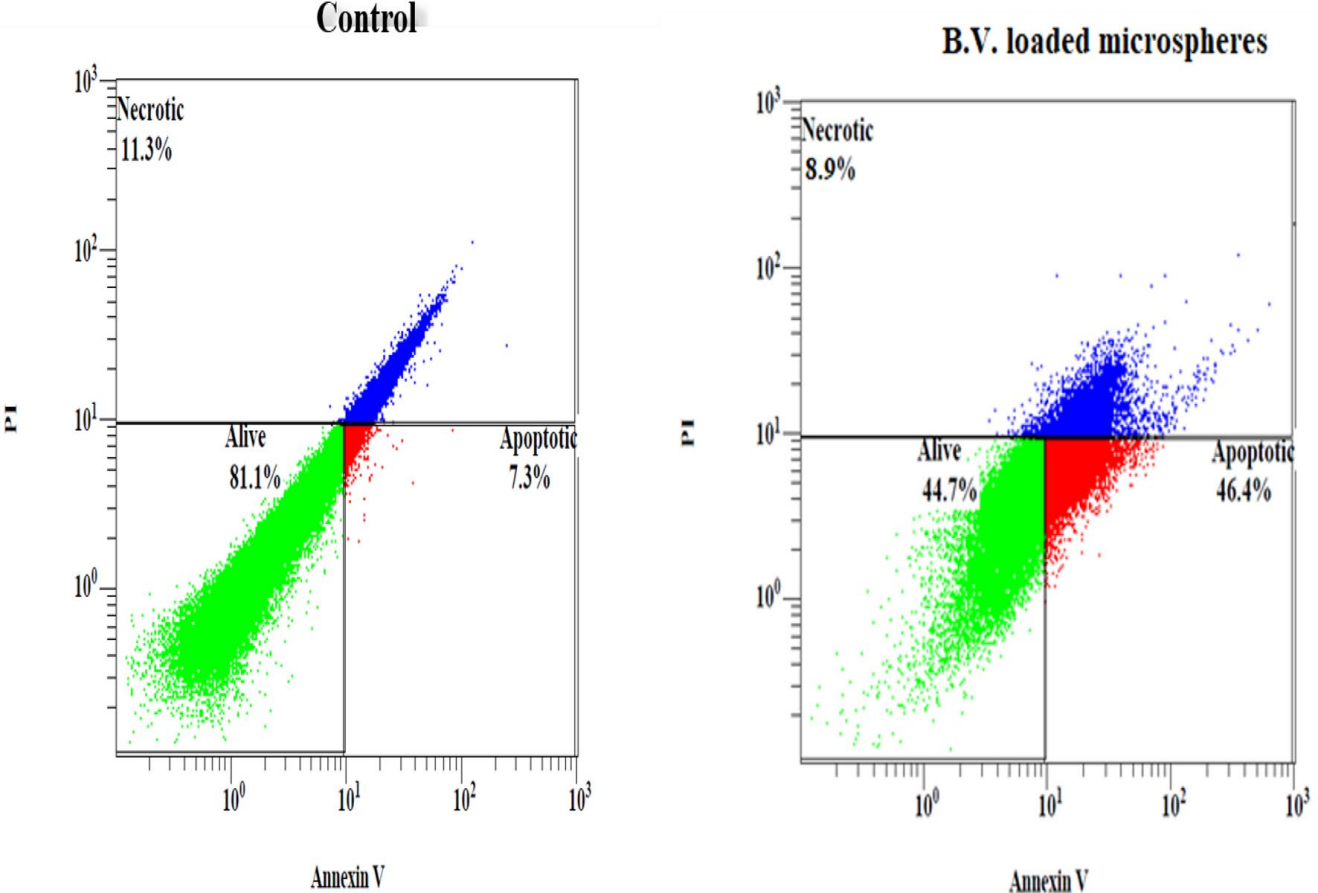
Flowcytometric analysis of PC3 cells treated with the 50% inhibitory concentrations of B.V. loaded cross-linked chitosan coated microspheres for 24 h. Apoptotic cells: are strongly express Annexin V, Necrotic cells: strongly expressed Propidium iodide (PI), Alive cells: are negative for both Annexin V and PI.

Flow cytometry based on annexin V-FITC/PI was carried out to quantify the apoptotic, necrotic, or dead cells after treatment. Figure 4 shows that the IC_50_ concentrations of B.V. loaded cross-linked chitosan-coated microspheres significantly induced apoptosis percent (*P* < 0.001), compared to control in PC3 cells, so the apoptotic cell death proportion (annexin V-positive/PI-negative) increased from 7.30 ± 0.30% in untreated control cells to 46.40 ± 0.80% (Table 5) in the treated group. Moreover, treatment of PC3 with B.V. loaded cross-linked chitosan-coated microspheres resulted in a significant decrease (*P* < 0.05), of necrosis percent, when compared to control cells (Table 5). To raise the durability and reduce the cancer chemo-resistance, several studies were concentrated on apoptosis induction as a potential solution. Chemo-resistant ovarian cancer cells were destroyed effectively through activation of intrinsic apoptosis by honey bee venom and chrysin^33^**.** Also, it has been found significantly more apoptotic cells for honeybee venom (8.3 ± 1.9%) compared to control (4.8 ± 0.4%) in breast cancer, SUM159 cells^13^. In study of Moselhy et al. free bee venom was able to cause induction of apoptosis in PC3 cells with 9.78% when compared to 1.51% in control cells^31^. According to the previous study, our results revealed that B.V. loaded cross-linked chitosan-coated microspheres increased induction apoptosis percent in PC3 cancer cells more than free B.V.

**Table 5:** The distribution of necrotic, apoptotic and living cells incubated with IC_50_ concentrations of B.V. loaded cross-linked chitosan coated microspheres for 24 h.

Tested Parameters	Mean ± SD		
	Percent of apoptotic cells	Percent of necrotic cells	Percent of viable cells
B.V. loaded microspheres	46.40 ± 0.80	8.90 ± 0.23	44.70 ± 0.90
Control	7.30 ± 0.30	11.30 ± 0.64	81.1 ± 0.84
Probability	0.0001***	0.015*	0.0001***

*P < 0.05, **P < 0.01 and ***P < 0.001 show the significance of difference between mean viability of untreated (control) and treated (experimental) cells.

## Conclusion

In conclusion, this study shows that prostate targeting B.V. loaded cross-linked chitosan microspheres can be successfully formulated with emulsion cross-linking method and coated by solvent evaporation method using ES100. In-vitro drug release profiles of different formulations showed that with increasing the drug: polymer ratio (increased polymer concentration), the release of the drug decreased. The free B.V and the optimized microsphere formula were more effective for destroying prostate cancer than doxorubicin considered one of the anti-cancer drugs. But microspheres treatment did not affect the viability of normal oral epithelial cells. According to flow cytometric analysis the optimized microsphere formula induced apoptosis and reduced necrosis percent at IC_50_ concentration. These results revealed that B.V. loaded cross-linked chitosan-coated microspheres inhibit the growth of PC3 and have a degree of specificity for malignant cells which suggests it as potential candidates to be employed in the evolution of improved anticancer agents in the future.

## Data Availability

All data generated or analyzed during this study are included in this published article.
